# Factors Related to a Smoke-free Home Status: A Parental Report

**DOI:** 10.31557/APJCP.2021.22.6.1865

**Published:** 2021-06

**Authors:** Nirun Intarut

**Affiliations:** *Health Systems Science Unit, Faculty of Medicine, Mahasarakham University, Muang, Maha Sarakham, Thailand. *

**Keywords:** Concurrent use, alcohol drinking, tobacco smoking, household smoking ban

## Abstract

**Purpose:**

The household smoking ban is one potential strategy for reducing exposure to second-hand smoke at home. There is little information about whether concurrent alcohol and tobacco use are related to a smoking ban at home. This study aimed to examine the association between concurrent alcohol and tobacco use with the household smoking ban strategy as reported by the parents of schoolchildren.

**Methods:**

We used data from a cross-sectional study that surveyed schoolchildren at 9 schools (grades 6 to 8). A self-administered questionnaire was sent out to the parents of 1,335 schoolchildren. Household smoking ban status was reported by the parents. We used multiple logistics regression to investigate the association between the household smoking ban and alcohol and tobacco use adjusted for potential confounders.

**Results:**

The prevalence of a no-smoking ban in households was 51% (95% confidence interval: 48.4%, 53.8%). After adjusting for the number of smokers in a home and perceptions about the harm of exposure to second-hand smoke, the multiple logistics regression results showed that concurrent alcohol and tobacco use in the households (OR = 2.31; 95% CI: 1.66, 3.20) had a higher risk of a no-smoking ban.

**Conclusions:**

Our findings showed that concurrent alcohol and tobacco use was associated with a smoking ban status in households. A no-alcohol-drinking-at-home campaign should be adopted and promoted for increasing the rate of smoke-free homes.

## Introduction

Exposure to second-hand smoke (SHS) is a factual cause of morbidity and mortality, especially in children (Max et al., 2012a; Carreras and Gorini, 2017). Places where children and non-smokers are frequently exposed to SHS include public places, cars, worksites, and homes (Antunes et al., 2016; Zheng et al., 2017; Tsai et al., 2018). 

Living in a smoke-free home is one of the strategies that can be employed to reduce exposure to SHS among children (Max et al., 2012b; Zhou et al., 2019). 

The influence of smoke-free home interventions in reducing SHS exposure at home is well documented (Behbod et al., 2018; Zhou et al., 2019). A home is a place where family members spend time talking, meeting, and carrying out various activities. A report from Bhutan found that 92.4% of participants exclusively drink only in the home. In Thailand, a study (Subady et al., 2013) reported that 43.1% and 34.9% of vocational school students had alcohol consumption parties, at home or in a dormitory, respectively. In addition, a report showed that most of the Thai population consumed alcoholic products at their own home, a friend’s home or a relative’s home (Assanangkornchai et al., 2010). Other studies (Drobes, 2002; Twyman et al., 2016) have shown a relationship between alcohol consumption and smoking. From this, however, it seems there is scarce evidence to show the association between concurrent alcohol and tobacco use (CATU) and no-smoking bans in households based on the perspectives of the parents of schoolchildren.

This study aimed to test the association between concurrent alcohol and tobacco use and the banning of smoking at home.

## Materials and Methods

In this investigation, we used data from a school-based study that aimed to explore the prevalence of exposure to SHS (SHS) among schoolchildren. This study used a cross-sectional school-based study conducted using nine randomly selected schools located throughout northeast Thailand during 2018-2019. 

At each school, we asked students in grades 6 to 8 to participate in the study. Of nine schools, a total of 2,278 students were recorded in a database. For each selected class, the trained research team collected data by using a self-administered questionnaire. We excluded students who were staying in the same home as another student. The parents and household variables were reported by a self-administered questionnaire. Students delivered the sealed questionnaire and consent form to their parents (mother, father, or relation). After they completed the questionnaires, the students returned them to school. We received questionnaires from 1,335 households (response rate: 58.6%; 1,335/2,278). Participants gave written informed consent to participate in the study. In this study, we used the parents’ data for testing our above-mentioned hypothesis. A total of 1335 parents were included in the study, and their data analysed.

*Variables and measurement*

A household smoking ban was assessed by asking parents the question: “Currently, do you allow smoking in the home?” Response options were (1) “no, not at all”, (2) “sometimes allow”, or (3) “allow”. In this analysis, we categorized this variable into two groups as follows: “smoking ban” refers to (1); “no smoking ban” refers to (2) and (3).

Perception about the harm of exposure to SHS was measured by this question: “Inhaling tobacco smoke negatively affects the health of infants and children. From this sentence, do you agree?” Response options were (1) “definitely disagree”, (2) “disagree”, (3) “agree”, or (4) “definitely agree”.

CATU was measured by two questions. The first question was: “In the past 1 month, has there been any alcohol consumption in your home?” Response choices were (1) “yes” or (2) “no”. The 2nd question was: “Has there been tobacco use?” Answer choices were (1) “yes, always smoking”, (2) “yes, sometimes smoking”, and (3) “no”. CATU at home was defined by dividing the questions as follows: (0) no (original code 2 in 1st question); (1) only tobacco smoking (original code 2 in the 1st question and 1, 2 in the 2nd question); (2) only alcohol drinking (original code 1 in the 1st question and 3 the 2nd question); (3) both (original code 1 in the 1st question and 1, 2 in the 2nd question).

Demographic characteristics were also used, as follows: age in years (≤ 40, > 40), gender (female, male), marital status (live with spouse/partner, divorced/widow), occupation (unemployed, civil service, agriculture, merchant), duration of school attendance by the guardian (0 years, 1-6 years, ≥ 7 years), household income per month (<10,000 baht, ≥ 10,000 baht), number of smokers in the house (none, 1, ≥2 persons).

*Statistical analysis*

Demographic characteristics were reported for a smoking ban. Univariate analysis used the Chi-square test or Fisher’s exact test, as appropriate. We selected the variables with p < 0.20 for multivariate analysis. For multivariate analysis, we used multiple logistics regression and adjusted for potential confounders. For model fitting, a backward elimination method was used to determine the final model. All data analysis was performed using R version 3.6.0 (R Core Team, 2013).

## Results

A total of 1335 participants were included in the analysis. The prevalence of a no-smoking ban in the home was 51.1% (95%CI: 48.4, 53.8). Table 1 shows the distribution of participants. Around 59.9% of participants were aged less than 40 years. Most parents (95.4%) who responded to the questionnaire were females. Most participants worked in agricultural occupations (83.9%), had attended school for 1-6 years (93.0%), and had a household income of less than 10,000 THB. 73.3% had one smoker in the home, while 33.0% disagreed with the perception about the harm of SHS exposure, and 36.6% were only tobacco users.

Univariate results are shown in Table 1. Statistically significant factors were as follows: the number of smokers in the house, perception about the harm of SHS exposure, and parental co-use of alcohol and tobacco. Not statistically significant factors were as follows: age, gender, marital status, occupation, duration of school attendance, and household income per month.

Table 2 shows the results of multiple logistics regression. Homes that only had one smoker (OR: 1.9; 95%CI: 1.4, 2.5) and more than one resident (OR: 1.6; 95%CI: 1.2, 2.2) had a higher risk of a no-smoking ban than that of no smokers in the home. Parents who agreed (OR: 1.9; 95%CI: 1.4, 2.5) and strongly agreed (OR: 1.6; 95%CI: 1.2, 2.2) with the risk perception of SHS exposure had a higher risk of no-smoking bans. Our findings showed that parents who had reported CATU in the home were 2.3 times more likely to have no-smoking bans compared to those who did not CASU in the home (OR = 2.3; 95% CI: 1.7, 3.2).

**Table 1:** Characteristics and Univariate Analysis of Participants

	Total n= 1335	Smoking Ban n= 653	No smoking Ban n= 682	P value
Age (Years)				0.405
≤ 40	757 (59.9)	341 (58.7)	416 (61)	
> 40	506 (40.1)	240 (41.3)	266 (39)	
Gender				0.434
Female	1273 (95.4)	557 (95.9)	716 (95)	
Male	62 (4.6)	24 (4.1)	38 (5)	
Marital status				0.55
Live with spouse/part	1076 (80.6)	464 (79.9)	612 (81.2)	
Divorced/widow	259 (19.4)	117 (20.1)	142 (18.8)	
Occupation				
Unemployed	72 (5.7)	36 (6.2)	36 (5.3)	0.409
Civil service	36 (2.9)	21 (3.6)	15 (2.2)	
Agriculture	1060 (83.9)	480 (82.6)	580 (85)	
Merchant	95 (7.5)	44 (7.6)	51 (7.5)	
Duration of school attendance by guardian(s) (years)				0.311
0	17 (1.3)	10 (1.7)	7 (1)	
1-6	1175 (93)	534 (91.9)	641 (94)	
≥ 7	71 (5.6)	37 (6.4)	34 (5)	
Household income per month (Thai baht)				0.271
<10,000	1078 (85.4)	489 (84.2)	589 (86.4)	
≥ 10,000	185 (14.6)	92 (15.8)	93 (13.6)	
Number of smokers in house (persons)				< 0.001
None	428 (33.9)	250 (43)	178 (26.1)	
1	547 (43.3)	207 (35.6)	340 (49.9)	
≥2	288 (22.8)	124 (21.3)	164 (24)	
Perception about the harm of eSHS				< 0.001
Definitely disagree	284 (21.3)	100 (17.2)	184 (24.4)	
Disagree	451 (33.8)	140 (24.1)	311 (41.2)	
Agree	299 (22.4)	175 (30.1)	124 (16.4)	
Definitely agree	301 (22.5)	166 (28.6)	135 (17.9)	
Concurrent alcohol and tobacco use				< 0.001
No	266 (22.4)	147 (27)	119 (18.4)	
Only tobacco use	436 (36.6)	238 (43.8)	198 (30.7)	
Only alcohol drinking	80 (6.7)	36 (6.6)	44 (6.8)	
Both	408 (34.3)	123 (22.6)	285 (44.1)	

**Table 2:** The Multiple Logistic Regression Results of Factors Associated to No Smoking Ban in Home

Variables	Crude OR (95%CI)	Adjusted OR (95%CI)
Number of smokers in house (persons)		
None	1	1
1	2.1 (1.6, 2.8)	1.9 (1.4,2.5)
≥2	1.9 (1.4, 2.5)	1.6 (1.2,2.2)
Perception about the harm of eSHS		
Strong disagree	1	1
Disagree	1.3 (0.9, 1.8)	1.4 (0.9, 1.9)
Agree	0.4 (0.3, 0.6)	0.4 (0.3, 0.6)
Strongly agree	0.5 (0.4, 0.7)	0.5 (0.4, 0.8)
Concurrent alcohol and tobacco use		
No	1	1
Only tobacco use	1.0 (0.8, 1.4)	0.9 (0.7, 1.2)
Only alcohol drinking	1.4 (0.9, 2.3)	1.4 (0.8, 2.3)
Both	2.7 (1.9, 3.7)	2.3 (1.7, 3.2)

## Discussion

Our findings show the high prevalence of no-smoking bans in homes. The following factors were associated with no-smoking bans in the home: having a smoker in the home, perception of the harm of SHS exposure, and CATU in the home.

The prevalence of homes without a smoke-free rule was reported at 88.5% (Antunes et al., 2016). In Europe, the prevalence varied from 20.2% in Romania to 45.6% in Spain (Fu et al., 2018). Such a difference in prevalence might be due to differences in population and study sample. Our study aimed to ask parents who looked after their child or children. Further, the study site was located in the northeast region of Thailand. This result is similar to a study from Canada, which found that homes with smokers tended to show that those smokers smoked inside the homes (Gregoire et al., 2016). 

For the perception of parental risk concerning SHS exposure, this is one of the key factors to consider banning smoking in the home. Our findings showed that parents who agreed with the risks of SHS exposure were more likely to ban smoking in the home than those who did not agree. This result is similar to a study that reported parents who have a high perception of parental risk concerning SHS exposure tended to refrain from smoking in the home(Myers et al., 2020). 

Our findings revealed that a statistical significance of CATU had a higher risk of no ban on smoking in the home than those who did not have CATU. Studies show the effects of alcohol consumption and tobacco use are linked to diseases (Bobo and Husten, 2000; Harrison and McKee, 2008; Halperin et al., 2010). The evidence also shows alcohol consumption is related to smoking (Grucza and Bierut, 2006; Soh et al., 2017). Therefore, a campaign for preventing or impeding alcohol consumption in the home may reduce the prevalence of smoking in the home.

Our study faced certain limitations. The results may not be generalized to the overall population because we carried out the study in a rural area of northeast Thailand. We used data from a cross-sectional study design and were reliant on self-reported data. Therefore, information bias may have occurred. Further, we were unable to identify a causal relationship between CATU and no ban on smoking in the home.

In conclusion, our findings show that concurrent alcohol and tobacco use tends to be associated with smoking ban status in the home. We also found agreement with the perception of the risks of SHS exposure was greater to ban smoking, and that having a smoker in the home was less to ban smoking in the home than those who had not. No alcohol consumption in the home campaigns should be adopted and promoted to increase the percentage of smoke-free homes.

## Author Contribution Statement

NI contributed to the design, implementation, writing, and finalization of the manuscript.

## References

[B1] Antunes H, Precioso J, Araujo AC (2016). Prevalence of secondhand smoke exposure in asthmatic children at home and in the car: A cross-sectional study. Rev Port Pneumol.

[B2] Assanangkornchai S, Sam-Angsri N, Rerngpongpan S (2010). Patterns of alcohol consumption in the Thai population: Results of the National Household Survey of 2007. Alcohol Alcohol.

[B3] Behbod B, Sharma M, Baxi R (2018). Family and carer smoking control programmes for reducing children’s exposure to environmental tobacco smoke. Cochrane Database Syst Rev.

[B4] Bobo JK, Husten C (2000). Sociocultural influences on smoking and drinking. Alcohol Res Health.

[B5] Carreras G, Gorini G (2017). Attributable mortality and morbidity to second-hand smoke in Europe. Tob Prev Cessation.

[B6] Drobes DJ (2002). Concurrent alcohol and tobacco dependence - Mechanisms and treatment. Alcohol Res Health.

[B7] Fu M, Castellano Y, Tigova O (2018). Prevalence and correlates of different smoking bans in homes and cars among smokers in six countries of the EUREST-PLUS ITC Europe Surveys. Tob Induc Dis.

[B8] Gregoire B, Azagba S, Asbridge M (2016). Smoke-free homes, smoking susceptibility and familial smoking among never-smoking high school students: a cross-sectional analysis. CMAJ Open.

[B9] Grucza RA, Bierut LJ (2006). Cigarette smoking and the risk for alcohol use disorders among adolescent drinkers. Alcohol Clin Exp Res.

[B10] Halperin AC, Smith SS, Heiligenstein E (2010). Cigarette smoking and associated health risks among students at five universities. Nicotine Tob Res.

[B11] Harrison EL, McKee SA (2008). Young adult non-daily smokers: patterns of alcohol and cigarette use. Addict Behav.

[B12] Max W, Sung HY, Shi Y (2012a). Deaths from secondhand smoke exposure in the United States: economic implications. Am J Public Health.

[B13] Max W, Sung HY, Shi YL (2012b). Exposure to Secondhand Smoke at Home and at Work in California. Public Health Rep.

[B14] Myers V, Rosen LJ, Zucker DM (2020). Parental Perceptions of Children’s Exposure to Tobacco Smoke and Parental Smoking Behaviour. Int J Environ Res Public Health.

[B15] Soh AZ, Chee CB, Wang YT (2017). Alcohol drinking and cigarette smoking in relation to risk of active tuberculosis: prospective cohort study. BMJ Open Respir Res.

[B16] Subady BN, Assanangkornchai S, Chongsuvivatwong V (2013). Prevalence, patterns and predictors of alcohol consumption in a mountainous district of Bhutan. Drug Alcohol Rev.

[B17] Tsai J, Homa DM, Gentzke AS (2018). Exposure to Secondhand Smoke Among Nonsmokers - United States, 1988-2014. MMWR Morb Mortal Wkly Rep.

[B18] Twyman L, Bonevski B, Paul C (2016). Factors associated with concurrent tobacco smoking and heavy alcohol consumption within a socioeconomically disadvantaged Australian sample. Subst Use Misuse.

[B19] Zheng ZL, Deng HY, Wu CP (2017). Secondhand smoke exposure of children at home and prevalence of parental smoking following implementation of the new tobacco control law in Macao. Public Health.

[B20] Zhou YH, Mak YW, Ho GWK (2019). Effectiveness of interventions to reduce exposure to parental secondhand smoke at home among children in China: A Systematic Review. Int J Environ Res Public Health.

